# Comparative Research on the Thermophysical Properties of Nano-Sized La_2_(Zr_0.7_Ce_0.3_)_2_O_7_ Synthesized by Different Routes

**DOI:** 10.3390/nano12142487

**Published:** 2022-07-20

**Authors:** Yue Wang, Bohuai Shao, Boyan Fu, Binglin Zou, Chunjie Wang

**Affiliations:** 1 College of Physical Science and Technology, Bohai University, Jinzhou 121013, China; wangsuiyue@foxmail.com (Y.W.); s610650883@163.com (B.S.); fby326xxx@163.com (B.F.); 2 State Key Laboratory of Rare Earth Resources Utilization, Changchun Institute of Applied Chemistry, Chinese Academy of Sciences, Changchun 130022, China; zoubinglin@ciac.ac.cn

**Keywords:** La_2_(Zr_0.7_Ce_0.3_)_2_O_7_, thermophysical performance, synthetic route, nano-sized, micro-strain

## Abstract

La_2_(Zr_0.7_Ce_0.3_)_2_O_7_ has been regarded as an ideal candidate for the next generation of thermal barrier coatings (TBCs) due to its prominent superiority. In this paper, the nano-sized La_2_(Zr_0.7_Ce_0.3_)_2_O_7_ was synthesized using two different synthetic routes: sol-gel and hydrothermal processes. Various techniques were utilized to assess the differences in the relevant thermophysical properties created by the different synthetic methods. According to the investigations, both samples exhibited pyrochlore structures with an excellent thermal stability. The sample synthesized via the hydrothermal method showed a more uniform particle size and morphology than that obtained through the sol-gel technique. The former also possessed a better sinter-resistance property, a more outstanding TEC (thermal expansion coefficient) and thermal conductivity, and a larger activation energy for crystal growth than the latter. The micro-strain of both samples showed an interesting change as the temperature increased, and 1200 °C was the turning point. Additionally, relative mechanisms were discussed in detail.

## 1. Introduction

For TBCs, a low thermal conductivity and sintering rate, a good phase stability and adherence, as well as compatibility with the thermal expansion coefficient (TEC) are typical criteria [1,2,3]. Recently, as a novel material for TBCs, La_2_(Zr_0.7_Ce_0.3_)_2_O_7_ (LZ7C3) with matching parameters has elicited people’s wide concern and consideration. The significance of LZ7C3 has been certified and reviewed through a significant amount of research, whether in powder or in coating form. Yin et al. studied the hot corrosive behavior of La_2_(Zr_0.7_Ce_0.3_)_2_O_7_ using a salt mixture (Na_2_SO_4_ + V_2_O_5_) [4]. Che et al. devoted their work to the investigation of the structural and thermophysical properties of La_2_(Zr_0.7_Ce_0.3_)_2_O_7_ through a molecular dynamic simulation, as well as through other experiments [5]. Wang et al. prepared nano-sized La_2_(Zr_0.7_Ce_0.3_)_2_O_7_ using the hydrothermal method and evaluated its relevant performance compared with 8YSZ and La_2_Zr_2_O_7_ [6]. Such previous studies have demonstrated that La_2_(Zr_0.7_Ce_0.3_)_2_O_7_ is a promising material for the application of TBCs. 

In fact, the performance of the powders/coatings is not only reliant upon their structures and components but also bound by their morphologies and particle sizes; thus, selecting a suitable synthesis method is essential to improving TBC properties. Furthermore, relevant reports have identified that some properties of TBC materials are influenced by extrinsic and intrinsic factors; for instance, the sintering rate is closely related to the activation energy for crystal growth (AECG), the micro-strain (MS) limits crystal growth behavior, and the chemical factors (such as adsorbed atoms) can change the surface energy and influence the correlation properties further [7]. Additionally, such factors are more or less affected by synthetic routes, therefore implying that the influence of synthetic processes is not ignorable. 

Nano-sized particles, on the other hand, differ obviously from their bulk counterparts due to their highly specific surface area, their greater grain boundaries, and their quantum effect. The larger S_BET_ (specific surface area) leads to a faster reaction rate and enhances the strength properties, the grain boundary is well correlated to the TEC of the material [8], and the quantum effect is conducive to improving the relevant natures [9], implying that the thermal as well as mechanical properties of the nano-sized materials notably improved in comparison with their corresponding bulk counterparts. At this point, various synthesis methods have been developed in order to obtain nanopowders with different particle diameters, size distributions, and morphologies. Among these methods, the hydrothermal and sol-gel techniques stand out due to their main advantages: low cost, simplicity, and ability to control the parameters of the reaction [10,11]. To those authors’ knowledge, nevertheless, there is a lack of information concerning the influences of the synthetic processes on the thermophysical properties of La_2_(Zr_0.7_Ce_0.3_)_2_O_7_. Since the physicochemical properties of powders are controlled by the reaction environment and the nucleation process, adopting an advisable method in order to create high-performance powders is important. 

The present study looks at the systematic comparisons, as described here, for La_2_(Zr_0.7_Ce_0.3_)_2_O_7_. The study also looks at the effects of the synthetic processes (sol-gel and hydrothermal processes) on the thermophysical properties, such as the phase structure, the thermal conductivity, TEC, the anti-sintering properties, the micro-strain, AECG, etc., as well as their related mechanisms that are also discussed in detail.

## 2. Materials and Methods

The sol-gel and hydrothermal processes have been employed to prepare La_2_(Zr_0.7_Ce_0.3_)_2_O_7_ nanocrystals with ZrOCl_2_·8H_2_O, La(NO_3_)_3_·6H_2_O, and Ce(NO_3_)_3_·6H_2_O (99.9%) as precursors. All of the chemicals were purchased from Guangdong Chenghai Chemicals Ltd. (Guangdong, China) without further purification.

(1) Sol-gel process
  Rare earth salts were dissolved in deionized water in order to form a stoichiometric mixture solution (C_total_ = 0.1 mol/L). Citric acid was added in a 1.2:1 molar ratio with respect to the metal ions. The solution was stirred at 80 °C and held at room temperature in a static condition for 12 h (for hydrolysis and condensation reactions), respectively. The products were vaporized at 90 °C and then turned into a porous body. After calcining at 1000 °C, La_2_(Zr_0.7_Ce_0.3_)_2_O_7_ can be obtained.
(2) Hydrothermal method
  A stoichiometric amount of the rare earth salt solution was mixed (C_total_ = 0.1 mol/L) with a certain amount of CTAB (surfactant, 2 wt. %). Sodium hydroxide (2M) was added in order to adjust the pH of the solution (to 9). Nano-sized La_2_(Zr_0.7_Ce_0.3_)_2_O_7_ can be obtained after heat-treating the above solution for 24 h based on the hydrothermal method at 200 °C. Finally, the precipitates were dried at 80 °C after they were washed 6 times with distilled water and ethanol.

In this study, LZ7C3 was prepared using these two synthetic routes, denoted as LZ7C3-SG and LZ7C3-HT. A thermogravimetric analyzer and differential scanning calorimeter (TG-DSC, Netzsch STA 449C Jupiter apparatus, Bavaria, Germany) and a X-ray diffractometer (XRD, Bruker D8, Karlsruhe, Germany) were used to evaluate the thermal behavior and phase structures of the samples, respectively. The FT-Raman spectra were investigated using a Thermo Nicolet 960 instrument. To study the thermophysical performance, some powders were compacted with a cold isostatic press (230 MPa) and calcined at different temperatures. The microstructure of the sample was estimated by scanning with an electron microscope (SEM, Hitachi S-4800, Tokyo, Japan). A high-temperature dilatometer (Netzsch 402 C, Bavaria, Germany) was employed for the investigation of TEC. The thermal conductivity was calculated and calibrated with Equations (1) and (2):

k = D_th_ρC_p_ (1)

k/k_0_ = 1−4Φ/3 (2)

where ρ, D_th_, C_p_, k_0_, and Φ are the density, thermal diffusivity, heat capacity, calibrated thermal conductivity, and segmental porosity, respectively. The specific heat capacity (C_p_) was calculated based on the chemical compositions of Ln_2_(Zr_0.7_Ce_0.3_)_2_O_7_ and the Neumann–Kopp rule [3]. 

## 3. Results and Discussion

**Figure 1:**
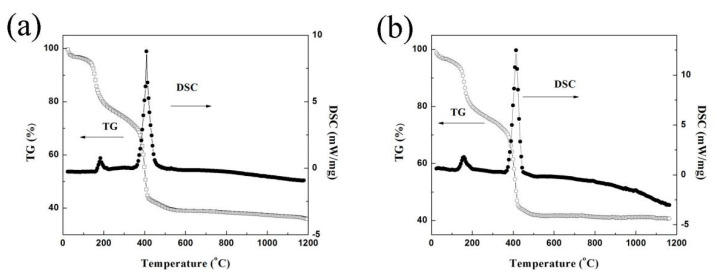
TG−DSC curves of dried powders: (**a**) LZ7C3−HT; (**b**) LZ7C3−SG.

Figure 1 presents the TG-DSC profiles of LZ7C3-HT and LZ7C3-SG from room temperature up to 1200 °C. Despite that these curves display a similar shape, some tiny distinctions can also be found. The total mass loss in LZ7C3-HT is 40–42%, which is slightly higher than that in LZ7C3-SG (39–41%). The major mass losses corresponding to the two remarkable exothermic peaks are around 195 and 423 °C for the DSC curves, which can be attributed to the removal of the water molecule and the decomposition of organic substances (citric acid, ethanol, and so on) [12,13]. No significant endothermic or exothermic peaks can be found above 550 °C for the TG and DSC curves, implying that such behaviors are completed. 

**Figure 2:**
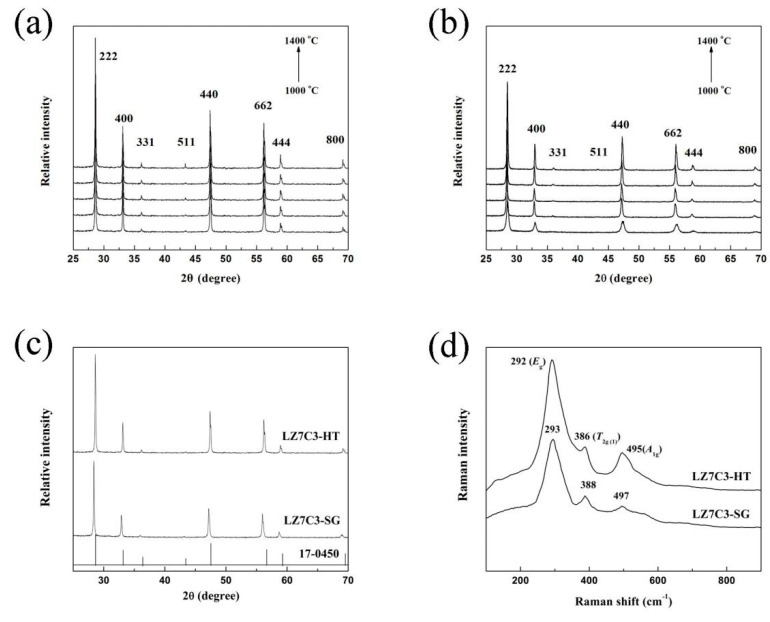
XRD patterns of LZ7C3−HT (**a**) and LZ7C3−SG (**b**) calcined at different temperatures; XRD patterns (**c**) and Raman spectra (**d**) of both samples calcined at 1300 °C.

Figure 2a,b give the XRD patterns of LZ7C3-HT and LZ7C3-SG powders calcined at various temperatures. All diffraction peaks of both samples are well matched with a pyrochlore structure (JCPDS 17-0450). The peaks around 28.7, 32.9, 35.9, 43.5, and 47.4° can be well indexed to the (222), (400), (331), (511), and (440) planes of the pyrochlore structure, respectively. No traces of other impurities can be detected in the whole test temperature range, suggesting that both samples possess an excellent thermal stability. Additionally, LZ7C3-HT displays better crystallinity than LZ7C3-SG, which can be attributed to the pressure in the autoclave generated during the heating reaction [14]. It is worth noting that, compared with LZ7C3-HT, the peak positions of LZ7C3-SG show a tiny blue shift (Figure 2c), implying a mismatch in lattice parameters between both samples. For this, the lattice parameters of LZ7C3-HT and LZ7C3-SG were further calculated, and the determined values were 10.81 and 10.84 Å, respectively. The Raman spectra of both samples sintered at 1300 °C were also investigated to further verify the phase structure, as shown in Figure 2d. It has been confirmed that rare earth zirconates with a pyrochlore structure possess six vibration modes, corresponding to the A_1g_, E_g_, and 4T_2g_ modes. In our cases, the spectra of both samples are remarkably similar with the exception of the peak intensity. The band located at 292 cm^−1^ belongs to the E_g_ mode, and the two residual bands around 386 and 495 cm^−1^ can be assigned to the 2T_2g_ mode. For the other three modes (A_1g_ and 2T_2g_), they are too weak at a normal pressure and are almost invisible [6]. In addition, no obvious characteristic peaks of other structures can be observed in both curves, meaning pure pyrochlore structures for both samples.

**Figure 3:**
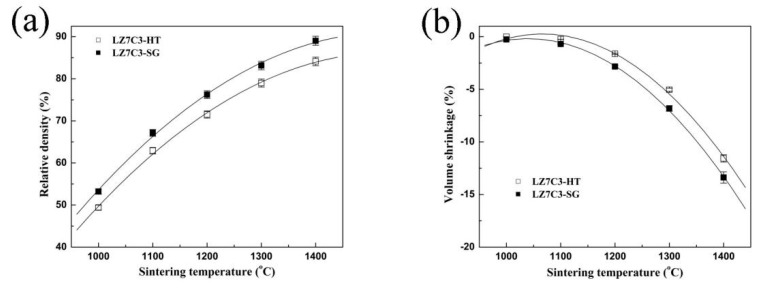
Relative densities (**a**) and volume shrinkages (**b**) of LZ7C3−HT and LZ7C3−SG bodies under different temperatures.

For a comparison of the thermophysical properties of LZ7C3-HT and LZ7C3-SG, the as-prepared powders were compacted at 230 MPa and then sintered at different temperatures. Figure 3 shows the relative densities and volume shrinkages of both samples, and the detailed measurement processes are described in Ref. [3]. The relative density of LZ7C3-HT is smaller than that of LZ7C3-SG, as seen in all of the test temperature points (Figure 3a). For both samples, the increase in relative density is obvious before reaching 1200 °C and then becomes gradual as the temperature further increases. At 1400 °C, the relative density determined for LZ7C3-HT is 83–84%, which is lower than that of LZ7C3-SG (88–89%). On the other hand, the volume shrinkage of both samples decreases simultaneously as the temperature increases, as shown in Figure 3b. Such results are consistent with the conclusion reported by Cao [15]. On the other hand, the volume shrinkage of LZ7C3-HT is 11–12% after heat-treatment at 1400 °C, while the corresponding value of LZ7C3-SG is 13–14%. 

**Figure 4:**
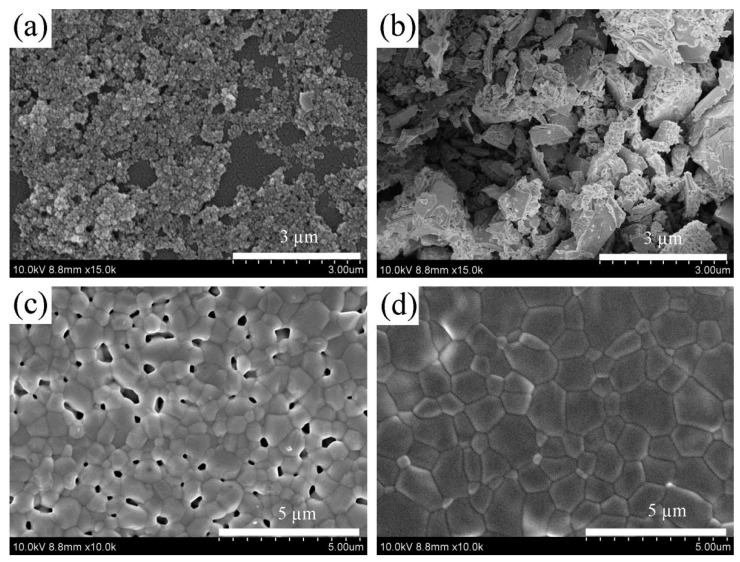
SEM images of as-prepared powders and compacted bodies calcined at 1400 °C of LZ7C3−HT (**a**,**c**) and LZ7C3−SG (**b**,**d**).

The SEM micrographs of the as-prepared powders and the surface of the compacted bodies of LZ7C3-HT and LZ7C3-SG calcined at 1400 °C are shown in Figure 4. Clearly, the selected synthetic methods have obvious effects on the morphologies of the samples (Figure 4a,b). LZ7C3-HT exhibits spherical particles with a uniform size, while LZ7C3-SG exhibits entirely different microstructures, and the polyhedron agglomerates are dominant. Moreover, the morphology of the compacted body surface can give us some information about the anti-sintering property of the material. As shown in Figure 4c, LZ7C3-HT shows a uniform particle size and well-defined boundaries compared with those of LZ7C3-SG. It is worth stressing that some micropores can also be observed on the surface of the body and are beneficial for improving the TEC of the coatings. Additionally, despite LZ7C3-SG (Figure 4d) possessing a denser body structure and larger particle size, no abnormal growths can be found and the boundaries are still clear. By contrast, LZ7C3-HT possesses a superior anti-sinterability than LZ7C3-SG.

As known to all, cation diffusion affects the densification/grain growth of materials [16]. The mainstream mechanisms include two aspects: the solute drag mechanism and the lattice distortion mechanism. For the former, the space charge layer can be formed at the grain boundary region, which can impede on the grain boundary migration. For the latter, the mismatch in ionic radii will lead to the distortion of the lattice [17]. As illustrated in Ref. [18], for the crystals, the local distortions give rise to the nonhomogeneous micro-strain that is accompanied by variations in some characteristics of XRD peaks, such as peak broadening. The micro-strain can bring the mobility of cations down and further affect the densification/grain growth of the materials [19]. Moreover, the different nucleation processes and the interactions between the inorganic precursor molecules during the nucleation processes that originate from different synthetic routes can also cause a mismatch in the micro-strain. At this point, the contributions of the crystal size and the micro-strain need to be considered. The Scherrer equation cannot work well for such cases and should be modified so the following relation can be obtained [20].

(3) $$\beta \cos\;\theta =\frac{k\lambda }{L}+4\varepsilon \;\sin\;\theta$$

**Figure 5:**
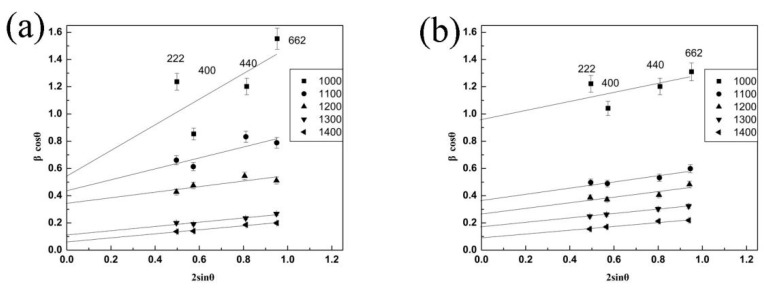
Plots of βcosθ vs. 2sinθ for various peaks (hkl) under different temperatures: (**a**) LZ7C3−HT; (**b**) LZ7C3−SG.

Here *β*, *θ*, *λ*, *L*, and *ε* are the full width half maximum, diffraction angle, wavelength of X-ray radiation, average size, and micro-strain, respectively, and K is set at 0.9, generally. Based on Equation (3), L and ε can be calculated based on the slope of *β*cos *θ* vs. 2sin *θ*. Figure 5 represents the relationship between *β*cos *θ* and 2sin *θ* with different peaks and temperatures, and 2*ε* (the slope of straight line) and kλ/L (intercept) can be obtained from the curves. For the micro-strain, the values of LZ7C3-HT determined from 1000 to 1400 °C are 0.4698 ± 0.0008, 0.2132 ± 0.0005, 0.0983 ± 0.0003, 0.0792 ± 0.0002, and 0.0747 ± 0.0001. Meanwhile, the corresponding values of LZ7C3-SG are 0.1647 ± 0.0008, 0.1215 ± 0.0004, 0.1027 ± 0.0003, 0.0839 ± 0.0002, and 0.0802 ± 0.0001. Additionally, the average crystal sizes (nm) of both samples are calculated and displayed in Table 1.

Generally, the micro-strain is mainly derived from local distortions of the atoms in the lattice, including dislocations, defects, and grain boundaries. As we know that for LZ7C3, La_2_O_3_ dissolves in ZrO_2_ and CeO_2_ crystal lattice by substitution solid solution, La^3+^ as the dopants, will be expelled and occupy the surface sites due to the “self-purification” mechanism, resulting in a reinforced grain boundary defect density and a large micro-strain. Thus, the grain boundary microstructure is the dominant contribution to the micro-strain in this study [21]. After annealing at a higher temperature however, the rearrangement of the local position of the atoms and the decrease in the density of the grain boundary defects will result in the release of the micro-strain. Consequently, the micro-strain decreases as the temperature increases. Interestingly, it should be noted from Figure 5, that the obtained values of both samples show different trends with 1200 °C as the inflection point. Below 1200 °C, the values of LZ7C3-HT are larger than those of LZ7C3-SG, which may be due to the faster nucleation based on the synthesis conditions of the hydrothermal method [22]. With a reinforced nucleation rate, La_2_O_3_ rapidly dissolves in ZrO_2_ and CeO_2_ crystals, and transfers to the surface sites, enhancing the grain boundary defect density, and brings a large micro-strain. Besides, the morphology of LZ7C3-HT is more uniform than that of LZ7C3-SG, According to Rush’s report [23], the high micro-strain with a small particle size may be closely bound with S_BET_ and the morphology of materials. On the other hand, for the high temperature zone, the micro-strain values of LZ7C3-HT and LZ7C3-SG are quite the opposite. From Table 1, it is noteworthy that the mean particle sizes of both samples exhibit the consistent trend with the micro-strain. Above 1200 °C, the average particle sizes of LZ7C3-SG are larger than those of LZ7C3-HT, and the mismatch becomes larger and larger, demonstrating that LZ7C3-SG possesses a more rapid growth rate than LZ7C3-HT. Nevertheless, a fast crystal growth rate will cause lattice distortion, forming more defects, and resulting in a larger micro-strain.

Furthermore, as stated in our previous studies [16,24], regarding the TBC materials, there are two competitive aspects in the crystal growth processes which are known as crystallization and sintering. Crystallization can impede the densification of materials, while sintering will lead to the degradation of its thermophysical performance due to the absence of micropores and microcracks. Thus, the activation energy of the crystal growth (AECG) of the TBC materials should be investigated in-depth. To this end, AECG of both samples were calculated based on Equation (4) with the mean crystal size (as shown in Table 1) obtained through Equation (3) [24]:

(4) $$D_{t}=D_{0}\times e^{\left(-Q/RT\right)}$$

**Figure 6:**
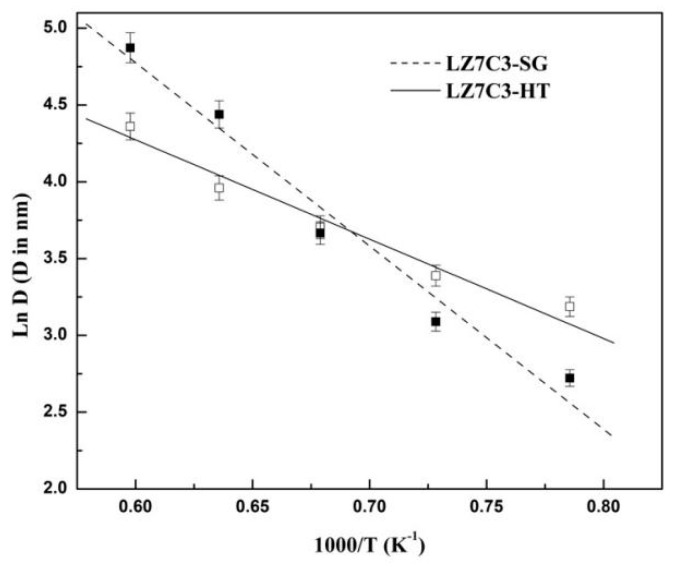
Relation between the crystal size and temperature plots of ln (*D*_t_/*D*_0_) against 1000/T.

Here, *D*_t_ and *D*_0_ correspond to the mean particle sizes for the initial and final temperatures, respectively. R is the gas constant, and T denotes the temperature. Based on Equation (4), the plot of Ln (*D*_t_/*D*_0_) vs. 1000/T for the two samples is exhibited in Figure 6, and AECG (Q) can be obtained from the slope. The values determined based on the above equation were found to be 99.31 ± 0.04 and 53.84 ± 0.05 KJ mol^−1^ for LZ7C3-HT and LZ7C3-SG, respectively. It is worth noting that the greater AECG means a slower crystal growth rate, which offers a better anti-resistance property for the materials. It is recognized that the crystal growth behavior is closely tied to defects, oxygen vacancies, micro-strain, and so on. For the La_2_(Zr_0.7_Ce_0.3_)_2_O_7_ system, La^3+^ will be expelled to the surface, enhancing the grain boundary defect density and micro-strain; both factors have substantial effects on the crystal growth behavior [25]. As mentioned above, LZ7C3-HT possesses a larger micro-strain than LZ7C3-SG below 1200 °C and the large micro-strain can impede the formation of necks between particles to form big particles, leading to a bigger AECG. On the other hand, above 1200 °C, based on studies by Leite et al. [26], who proposed a grain-rotation-induced grain coalescence model for the growth of nanocrystalline materials, assuming that the crystallographic orientations of the grains are in agreement, the rotation of the particles gives rise to a coherent interface among neighboring particles, allowing for single, large grains to form through the elimination of the grain boundaries of the particles. In our cases, in comparison with LZ7C3-SG, LZ7C3-HT exhibits smaller particle sizes and more regular morphologies, causing more complicated grain boundaries. To eliminate the grain boundaries in order to form big particles, more energy is required, and thus, the AECG of LZ7C3-HT is larger than that of LZ7C3-SG for high temperature ranges.

**Figure 7:**
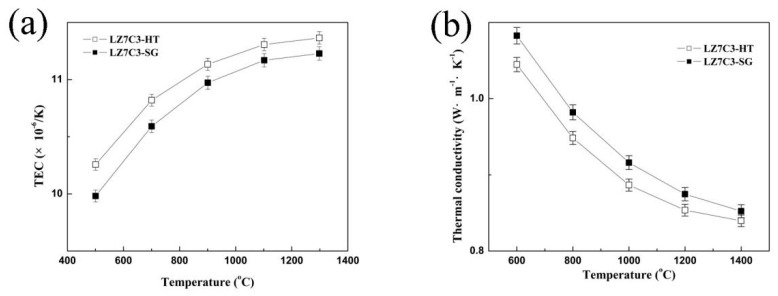
TECs (**a**) and thermal conductivities (**b**) of LZ7C3−HT and LZ7C3−SG as functions of temperature.

Figure 7 presents the thermal expansion coefficients and thermal conductivities of LZ7C3-HT and LZ7C3-SG under different temperatures, respectively. Obviously, the changes in both parameters show an opposing trend as the temperature increases. For each temperature point, LZ7C3-HT has higher TEC values than LZ7C3-SG. For example, the value of LZ7C3-HT at 1300 °C is 11.43 ± 0.06 × 10^−6^ K^−1^, while the corresponding value of LZ7C3-SG is 11.23 ± 0.05 × 10^−6^ K^−1^. This difference may be attributed to the small crystal size and the regular morphology of LZ7C3-HT. According to Rupp’s report [27], for nanocrystallites, the TEC is dependent upon the microstructure of the grain boundary. The larger the grain boundary defect density is, the more enhanced the TEC will be. The vibrational motion of the atom is sensitive to its surrounding coordinates. The enhanced static shift in the atoms and the bigger micro-strain lead to an increased TEC. On the other hand, the thermal conductivities determined for LZ7C3-HT and LZ7C3-SG are 0.81 ± 0.03 and 0.84 ± 0.02 W m^−1^ K^−1^, respectively.

## 4. Conclusions

Based on our findings, a direct relationship between the synthetic routes and thermophysical properties of La_2_(Zr_0.7_Ce_0.3_)_2_O_7_ can be observed. Both samples were synthesized via sol-gel and hydrothermal processes and belong to the pyrochlore type. No foreign structure can be found at even higher temperatures, indicating their exceptional thermostability. Compared with LZ7C3-SG, LZ7C3-HT yields a more uniform morphology and grain size, which have crucial roles in improving the thermophysical properties. LZ7C3-HT possesses a high TEC (11.43 ± 0.06 × 10^−6^ K^−1^) and a low thermal conductivity (0.81 ± 0.03 W m^−1^ K^−1^), while the values of LZ7C3-SG are 11.23 ± 0.05 × 10^−6^ K^−1^ and 0.84 ± 0.02 W m^−1^ K^−1^, respectively. The micro-strains of both samples show an interesting variation with 1200 °C as the inflection point. Due to the larger micro-strain and grain boundary effect, LZ7C3-HT exhibits a greater activation energy for crystal growth and better sinter-resistance properties than LZ7C3-SG.

## Figures and Tables

**Table 1:** Mean crystal sizes of both samples at different temperatures.

Sample Temp	1000 °C	1100 °C	1200 °C	1300 °C	1400 °C
LZ7C3-HT	24.66 ± 0.07	29.36 ± 0.06	38.42 ± 0.04	56.96 ± 0.02	92.43 ± 0.02
LZ7C3-SG	15.21 ± 0.05	21.96 ± 0.04	39.09 ± 0.02	84.70 ± 0.03	142.67 ± 0.02

## Data Availability

The data presented in this study are available from the corresponding author upon request.
